# Catalyzing PET‐RAFT Polymerizations Using Inherently Photoactive Zinc Myoglobin

**DOI:** 10.1002/anie.202414431

**Published:** 2024-10-29

**Authors:** Ian C. Anderson, Darwin C. Gomez, Meijing Zhang, Stephen J. Koehler, C. Adrian Figg

**Affiliations:** ^1^ Department of Chemistry and Macromolecular Innovation Institute Virginia Tech Blacksburg Virginia 24061 United States of America

**Keywords:** Biocatalysis, Photocatalysis, Polymerization, PET-RAFT, Reversible-deactivation radical polymerization

## Abstract

Protein photocatalysts provide a modular platform to access new reaction pathways and affect product outcomes, but their use in polymer synthesis is limited because co‐catalysts and/or co‐reductants are required to complete catalytic cycles. Herein, we report using zinc myoglobin (ZnMb), an inherently photoactive protein, to mediate photoinduced electron/energy transfer (PET) reversible addition‐fragmentation chain transfer (RAFT) polymerizations. Using ZnMb as the sole reagent for catalysis, photomediated polymerizations of *N*,*N*‐dimethylacrylamide in PBS were achieved with predictable molecular weights, dispersity values approaching 1.1, and high chain‐end fidelity. We found that initial apparent rate constants of polymerization increased from 4.6×10–5 s^−1^ for zinc mesoporpyhrin IX (ZnMIX) to 6.5×10–5 s^−1^ when ZnMIX was incorporated into myoglobin to yield ZnMb, indicating that the protein binding site enhanced catalytic activity. Chain extension reactions comparing ZnMb‐mediated RAFT polymerizations to thermally‐initiated RAFT polymerizations showed minimal differences in block copolymer molecular weights and dispersities. This work enables studies to elucidate how protein modifications (e.g., secondary structure folding, site‐directed mutagenesis, directed evolution) can be used to modulate polymerization outcomes (e.g., selective monomer additions towards sequence control, tacticity control, molar mass distributions).

## Graphical abstract

Photocatalyzed reversible addition‐fragmentation chain transfer (RAFT) polymerizations are achieved using photoactive zinc myoglobin without the need for any additional reducing agents or co‐catalysts. Zinc myoglobin shows improved catalytic activity over the free zinc porphyrin cofactor (zinc mesoporphyrin IX) and leads to controlled polymerizations with predictable molecular weights, low dispersities, and retention of chain‐end functional groups.

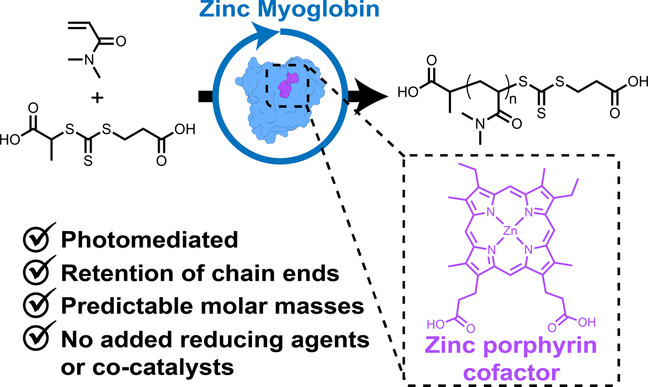

Photoinduced electron/energy transfer (PET) catalysts provide spatiotemporal control over reversible addition‐fragmentation chain transfer (RAFT) polymerizations, leading to discrete control over surface patterning,[^1^, ^2^, ^3^] toggling between reactive species (e.g., radical, cationic) according to light irradiation,[^4^, ^5^] and 4‐dimensional material growth.[^6^, ^7^] PET–RAFT polymerizations lead to fewer irreversible termination events and better control over polymer products compared to conventionally initiated radical polymerizations.[^8^, ^9^, ^10^] Moreover, water‐soluble PET catalysts enable modification of biomolecules or biological systems, including synthesis of polymer–protein conjugates,[^11^, ^12^] cell‐surface modifications,^[13]^ or RAFT polymerizations within cells.^[14]^ However, PET catalyst modifications are limited to a narrow range of functional group modifications (e.g., changing halogens, adding conjugation) to tune redox potentials and absorption profiles.[^15^, ^16^, ^17^]

Proteins are a modular platform to tune reactivity because they contain active sites where site‐directed mutagenesis and directed evolution can tune steric and electronic properties to favor specific synthetic outcomes (e.g., reagent selectivity,[^18^, ^19^] stereo control,[^20^, ^21^] novel bond‐forming reactions).[^22^, ^23^] While protein‐based synthetic catalysts have shown great success in small molecule synthesis, these types of challenging transformations remain a goal in polymer chemistry that protein‐based catalysts have yet to achieve.

Cofactors,[^24^, ^25^, ^26^] proteins,[^27^, ^28^, ^29^, ^30^, ^31^, ^32^] or bacterial redox cycles,[^33^, ^34^, ^35^, ^36^, ^37^, ^38^] along with exogenous reagents have been used to introduce radicals into polymerizations. For example, metal‐containing proteins (e.g., laccase,^[39]^ horseradish peroxidase,[^32^, ^40^, ^41^, ^42^, ^43^] myoglobin,^[44]^ hemoglobin)^[45]^ catalyze atom transfer radical polymerizations (ATRP) or initiate RAFT polymerizations but require a reducing agent to regenerate the catalyst and to control polymerizations. Tyrosine residues on proteins (e.g., bovine serum albumin) can initiate a photo‐RAFT polymerization but need high concentrations of proteins containing tyrosine.^[46]^

An and co‐workers used an enzyme for photo‐RAFT polymerizations.^[47]^ Glucose oxidase (GOx), in combination with glucose, initiated a RAFT polymerization under blue‐light irradiation. Critically, adding glucose to the reaction reduced GOx to the photoexcitable state. Next, excitation and electron transfer to monomers or the thiocarbonylthio (TCT) initiated polymerizations (Figure 1). While direct PET activation of the RAFT TCT can occur, GOx likely reduced a monomer to initiate a conventional RAFT polymerization since [monomer]≫[TCT].^[47]^ Inspired by this pioneering system, we envisioned a protein‐based PET catalyst that can mediate a RAFT polymerization without the need for additional reagents and without monomer‐derived initiation (Figure 1).

**Figure 1:**
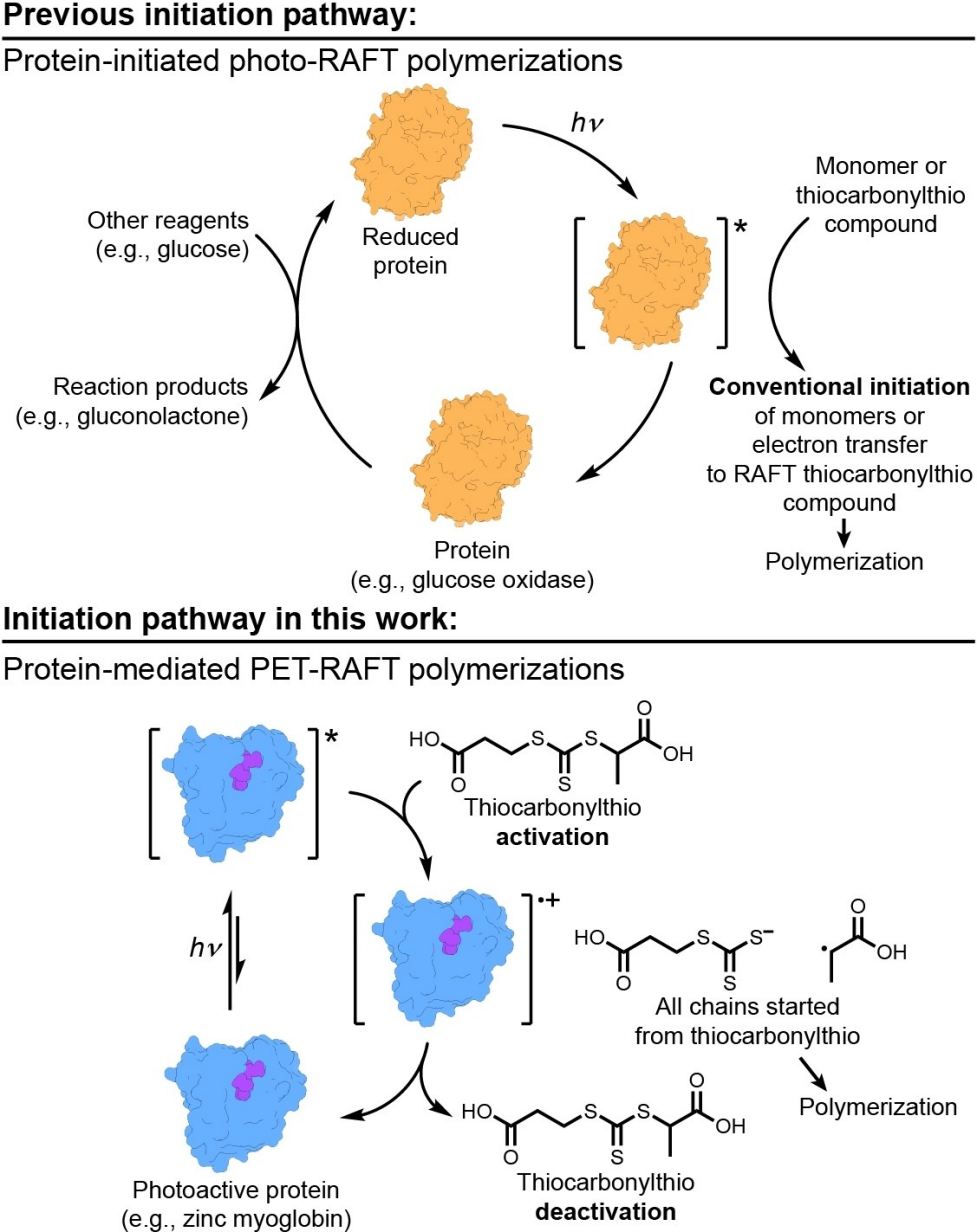
Previous work with enzymes as photocatalysts requires exogenous reagents for catalytic cycles, leading to conventional initiation of polymerizations.^[47]^ This work uses an inherently photoactive protein to catalyze radical polymerizations with no exogenous reagents.

Native iron myoglobin contains a non‐covalently bound heme cofactor that can be readily replaced with a synthetic zinc porphyrin to yield photoactive ZnMb.^[48]^ Gray's studies of photoredox properties of ZnMb showed that electron transfer readily occurs after photo excitation.^[49]^ Additionally, zinc mesoporphyrin IX (ZnMIX) is structurally similar to zinc tetraphenyl porphyrin, which commonly finds use in PET–RAFT catalysis.[^50^, ^51^] Therefore, we hypothesized that the inherent electron transfer properties of ZnMb can be used to mediate RAFT polymerizations upon photoexcitation, where no other reagents are required.

We prepared ZnMb by extracting native heme from myoglobin with a 1 : 1 butan‐2‐one/water mixture.^[48]^ The denatured protein, apo‐Mb, was re‐folded in PBS under highly dilute conditions and concentrated via spin‐filtration to yield apo‐Mb. ZnMIX was added dropwise over 16 h to a concentrated solution of apo‐Mb yielding ZnMb. Purification via preparatory size‐exclusion chromatography (SEC) removed unincorporated ZnMIX. UV/Vis spectroscopy shows a characteristic shift in the peak absorbance from 400 nm to 414 nm and the appearance of vibronic bands at 541 and 583 nm (**Figure S1**).^[48]^ Circular dichroism (CD) spectral analysis (CD Pro Software)^[52]^ revealed secondary structure changes in ZnMb compared to Mb (**Figure S2**). Distorted *α*‐helices, regular *β*‐strands, distorted *β*‐strands, turns, and un‐structured regions all changed by 8–13 %. These data suggested that ZnMb folded into defined secondary structures around the porphyrin but in a different metastable state compared to Mb.

To test whether ZnMb catalyzes RAFT polymerizations, we performed kinetic studies using *N*,*N*‐dimethylacrylamide (DMA) and 2‐[[(2‐Carboxyethyl)sulfanylthiocarbonyl]‐sulfanyl]propanoic acid as the chain transfer agent (CTA, Figure 2A). We tracked DMA conversion by ^1^H NMR spectroscopy monitoring the disappearance of a vinyl peak at *δ*=6.57 ppm in relation to an internal *N*,*N*‐dimethylformamide standard.

**Figure 2:**
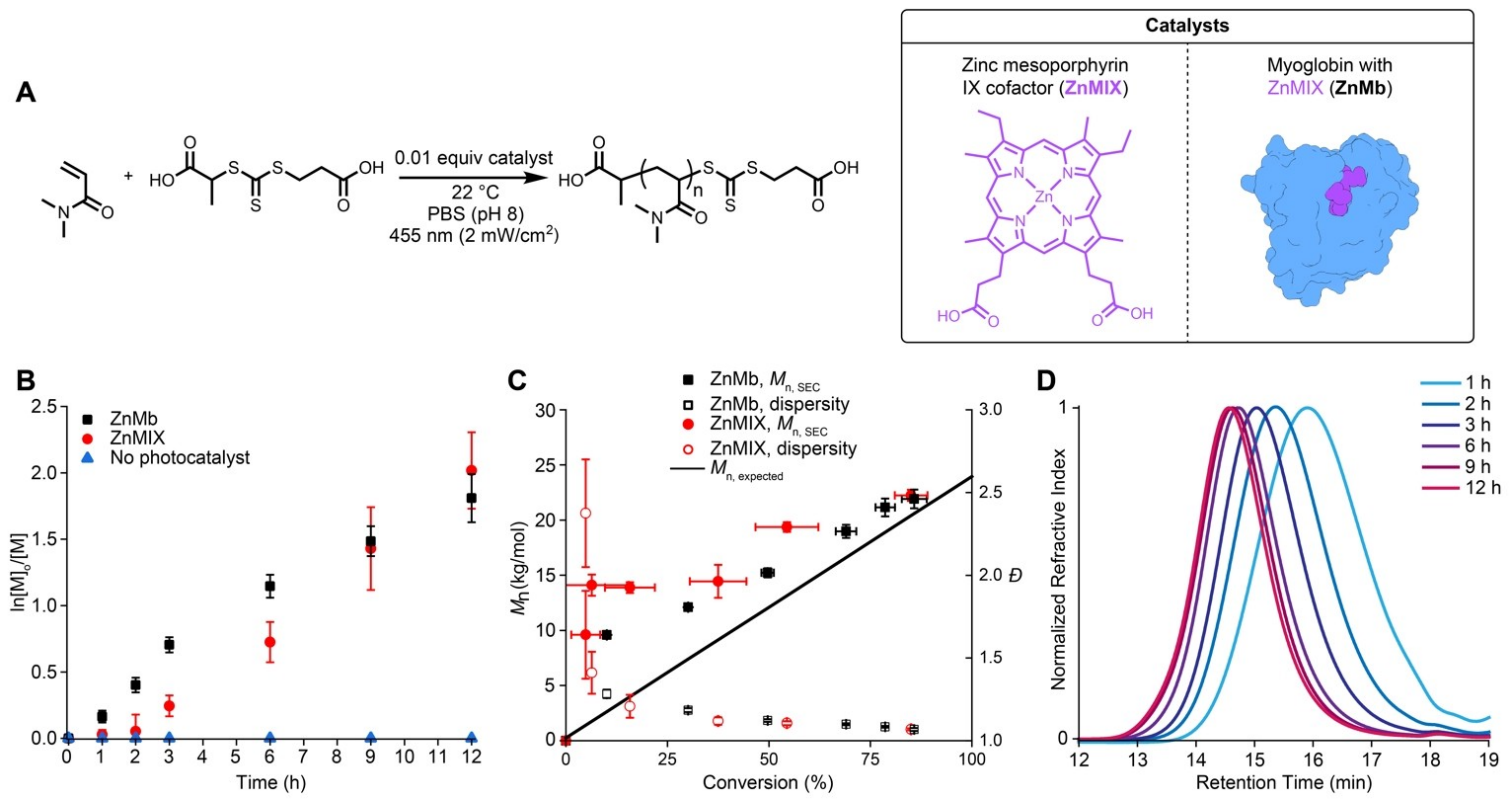
(A) Scheme of reversible addition–fragmentation chain transfer (RAFT) polymerization using different catalysts; pseudo‐first order kinetics plot (B) and molecular weight versus conversion plot (C) of polymerizations comparing different catalysts; (D) size‐exclusion chromatograms of polymerizations catalyzed by zinc myoglobin.

First, as CTAs can absorb blue light and undergo photoiniferter polymerizations,[^53^, ^54^] we irradiated solutions containing only DMA and CTA in PBS with 455‐nm light and monitored monomer conversion (Figure 2B). We observed minimal DMA conversion (<5 %) after 12 h as expected because of the lower monomer concentration (1 M vs. 2 M) and light source power (2 mW/cm^2^ vs. 16 mW/cm^2^) compared to previous reports.^[55]^ This control experiment supported our expectation that any observable polymerization would be attributed to ZnMIX or ZnMb.

With ZnMIX (0.01 equiv), CTA (1 equiv), and DMA (200 equiv), we observed linear pseudo first‐order kinetics with an apparent rate constant of polymerization (*k*_app_) of 4.6×10^−5^ s^−1^ (Figure S3) after an inhibition period of 2 h. Adding 5 v/v % DMSO only moderately improved the ZnMIX polymerization (Figure S4). Higher‐than‐expected polymer molar masses resulted (Figure 2C). However, we obtained predictable polymer molar masses showing low dispersity later in the reaction. These results show that ZnMIX can be a PET–RAFT catalyst but result in slow polymer chain initiation.

With ZnMb (0.01 equiv), CTA (1 equiv), and DMA (200 equiv), we obtained distinct kinetics compared to those with ZnMIX (Figures 2B, S5)—no inhibition and *k*_app_=6.5×10^−5^ s^−1^. The *k*_app_ of the ZnMb polymerizations decreased to 3.1×10^−5^ s^−1^ after 6 h. UV/Vis spectroscopy showed that the rate constant decrease was due to catalyst degradation because of a decrease in the Soret band of zinc porphyrin (Figure S6). Additionally, the increasing steric interactions between growing polymer chains and ZnMb likely contributes to the decrease in polymerization rate (vide infra). However, we observed no catalyst degradation in the ZnMIX polymerizations; indicating that decrease in polymerization resulted from a change in protein structure. We saw linear increases in polymer molar mass according to conversion and low dispersity samples with symmetric and monomodal size exclusion chromatograms (Figure 2C, 2D). The slow initiation, characterized by higher‐than‐expected molar masses, was less prominent compared to ZnMIX. Tacticity analysis^[56]^ showed atactic polymers (Figure S7). Overall, these result show that incorporating ZnMIX into Mb to yield ZnMb improved the catalytic efficiency by 1.4× early in the polymerizations but led to premature catalyst degradation, suggesting that the local protein environment affected catalytic activity.

To confirm photomediated polymerizations, we monitored DMA conversion by ^1^H NMR spectroscopy in the presence and absence of 455 nm light irradiation (Figure 3). We pulsed the light irradiation four times. Each time, we observed monomer conversion with irradiation and <2 % monomer conversion in the dark, which is within the error of ^1^H NMR spectroscopy. These results confirmed that ZnMb polymerizations were photomediated with little background catalysis in the absence of blue light.

**Figure 3:**
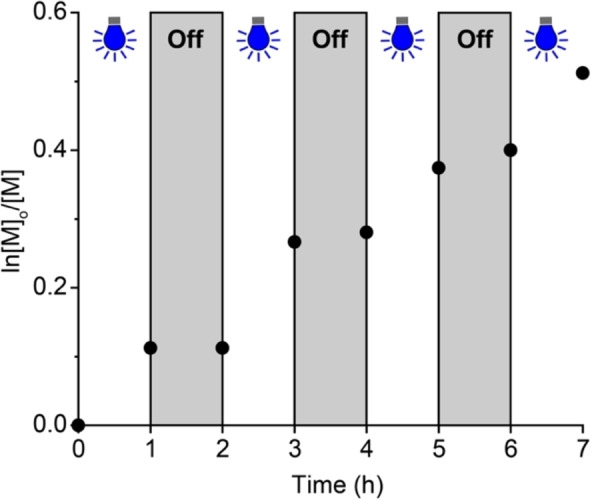
Experiments testing temporal control over a zinc myoglobin‐mediated reversible addition‐fragmentation chain transfer (RAFT) polymerization of *N*,*N*‐dimethylacrylamide.

Finally, we set up chain‐extension reactions to study the chain‐end retention of the CTA following ZnMb‐mediated PET–RAFT polymerizations (Figure 4). We prepared poly(*N*,*N*‐dimethylacrylamide) (PDMA) using ZnMb as the photocatalyst (*M*_n, expected_=11400 g/mol, *M*_n, SEC_=13,900 g/mol, *Ð*=1.19, Figure S8). We purified PDMA from excess protein by dialyzing against ultrapure water for 36 h, which precipitated out ZnMb. After centrifuging to separate the soluble fraction from any precipitates, UV/Vis spectroscopy indicated that a majority of the protein was removed (Figure S9).

**Figure 4:**
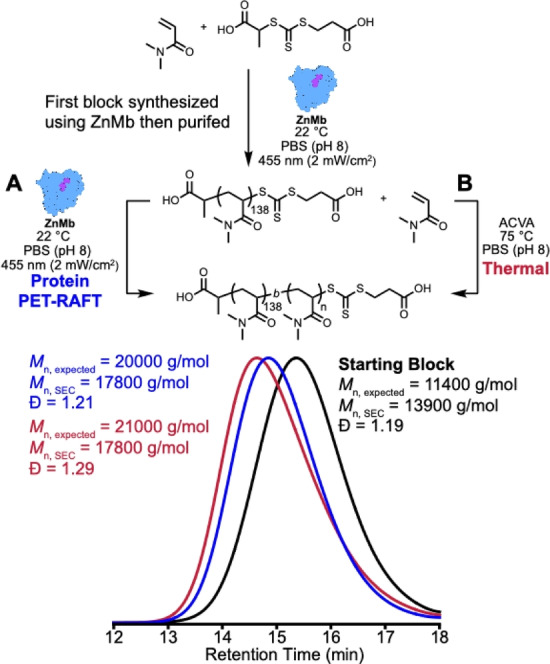
Chain extension polymerizations of a poly(*N,N*‐dimethylacrylamide) first block synthesized using zinc myoglobin (ZnMb) extended using zinc myoglobin as a photocatalyst (A) or 4,4′‐azobis(4‐cyanovaleric acid) thermal initiator (B).

We set up a PET‐RAFT polymerization to determine if ZnMb can catalyze the synthesis of a block copolymer (Figure 4A). We irradiated solutions for 17 h using 0.01 equiv. of ZnMB relative to PDMA and 100 equiv. of DMA, reaching 62 % monomer conversion by ^1^H NMR spectroscopy (Figure S10). We measured the molar mass of the block copolymer (*M*_n, SEC_=17800 g/mol) by size exclusion chromatography equipped with a multi‐angle light scattering detector. The result agreed with the expected molar mass from the monomer conversion (*M*_n, expected_=20000 g/mol) and a low dispersity was maintained (*Ð*=1.21). However, the polymerization reached a lower conversion in the same amount of time compared a polymerization of the first block. For example, kinetics experiments (Figure 2B) reached 80 % DMA conversion after 12 h compared to chain extensions only reaching 62 % DMA conversion after 12 h.

Since the ZnMb‐catalyzed chain extension was slower than expected, we set up a thermal chain‐extension polymerization of PDMA to compare block copolymers synthesized using ZnMb versus azo initiators. Chain extensions were run with 0.2 equiv. of 4,4′‐azobis(4‐cyanovaleric acid) (ACVA) and 65 equiv. of DMA at 75 °C for 17 h, reaching >95 % conversion of DMA by ^1^H NMR spectroscopy (Figure S11). SEC analysis showed a uniform shift to lower retention times, expected versus experimental molar masses were similar (*M*_n, expected_=21000 g/mol, *M*_n, SEC_=17800 g/mol), and a low dispersity was obtained (*Ð*=1.29). Therefore, PMDA block copolymers synthesized via PET‐RAFT and an ACVA initiator showed similar SEC profiles, resulting in no significant difference between either approach for synthesizing block copolymers.

The chain extension polymerizations suggested steric interactions between ZnMb and PDMA that slowed down the rate of polymerization. We expect that these steric interactions between the polymer chain end and protein catalyst resemble those of protein‐catalyzed ATRP instead of protein‐initiated RAFT polymerizations because the zinc porphyrin co‐factor must directly react with the CTA chain end.^[57]^ However, these interactions did not lead to unpredictable molar masses or increases in dispersity.

In conclusion, inherently photoactive ZnMb was used to catalyze PET–RAFT polymerizations. These results introduce an opportunity to study how photoactive protein structure can be used to control the synthesis of acrylic polymers. Additionally, polymerization kinetics showed the necessary qualities of a controlled polymerization. The catalytic efficiency of ZnMIX improved when incorporated into Mb, however we observed decreased catalyst activity at higher polymer molar masses. What is the effect of polymer molar mass on ZnMb catalytic efficiency? Can mutagenesis be performed on myoglobin to further increase the catalytic activity? More broadly, this first example of an inherently photoactive protein mediating a RAFT polymerization informs investigations into how to use proteins to synthesize acrylic polymers that more closely mimic proteins (e.g., selective monomer additions towards sequence control, tacticity control, etc.).

## Supporting Information

The authors have cited additional references within the Supporting Information.[^58^, ^59^]

## Conflict of Interests

The authors declare no conflict of interest.

## Supporting information

As a service to our authors and readers, this journal provides supporting information supplied by the authors. Such materials are peer reviewed and may be re‐organized for online delivery, but are not copy‐edited or typeset. Technical support issues arising from supporting information (other than missing files) should be addressed to the authors.

Supporting Information

## Data Availability

The data that support the findings of this study are available from the corresponding author upon reasonable request.

## References

[1] B. Hunter , K. Bell , C. W. Pester , ACS Appl. Polym. Mater. 2023, doi: 10.1021/acsapm.3c02460.

[2] M. Li , M. Fromel , D. Ranaweera , S. Rocha , C. Boyer , C. W. Pester , ACS Macro Lett. 2019, 8, 374–380. 35651140 10.1021/acsmacrolett.9b00089

[3] K. Lee , N. Corrigan , C. Boyer , Angew. Chem. Int. Ed. 2021, 60, 8839–8850. 10.1002/anie.202016523 33449437

[4] Y. Ma , V. Kottisch , E. A. McLoughlin , Z. W. Rouse , M. J. Supej , S. P. Baker , B. P. Fors , J. Am. Chem. Soc. 2021, 143, 21200–21205. 34878283 10.1021/jacs.1c09523

[5] B. Zhao , J. Li , Z. Li , X. Lin , X. Pan , Z. Zhang , J. Zhu , Macromolecules 2022, 55, 7181–7192.

[6] Z. Zhang , N. Corrigan , A. Bagheri , J. Jin , C. Boyer , Angew. Chem. Int. Ed. 2019, 58, 17954–17963. 10.1002/anie.201912608 31642580

[7] C. W. A. Bainbridge , K. E. Engel , J. Jin , Polym. Chem. 2020, 11, 4084–4093.

[8] M. L. Allegrezza , D. Konkolewicz , ACS Macro Lett. 2021, 10, 433–446. 35549229 10.1021/acsmacrolett.1c00046

[9] J. Xu , K. Jung , A. Atme , S. Shanmugam , C. Boyer , J. Am. Chem. Soc. 2014, 136, 5508–5519. 24689993 10.1021/ja501745g

[10] J. G. Baker , R. Zhang , C. A. Figg , J. Am. Chem. Soc. 2024, 146, 106–111. 38128915 10.1021/jacs.3c12221 PMC10785814

[11] A. Watanabe , J. Niu , D. J. Lunn , J. Lawrence , A. S. Knight , M. Zhang , C. J. Hawker , J. Polym. Sci. 2018, 56, 1259–1268.

[12] B. S. Tucker , M. L. Coughlin , C. A. Figg , B. S. Sumerlin , ACS Macro Lett. 2017, 6, 452–457. 35610863 10.1021/acsmacrolett.7b00140

[13] J. Niu , D. J. Lunn , A. Pusuluri , J. I. Yoo , M. A. O'Malley , S. Mitragotri , H. T. Soh , C. J. Hawker , Nat. Chem. 2017, 9, 537–545. 28537595 10.1038/nchem.2713

[14] Y. Zhang , Q. Gao , W. Li , R. He , L. Zhu , Q. Lian , L. Wang , Y. Li , M. Bradley , J. Geng , JACS Au 2022, 2, 579–589. 35373203 10.1021/jacsau.1c00373 PMC8970002

[15] E. Speckmeier , T. G. Fischer , K. Zeitler , J. Am. Chem. Soc. 2018, 140, 15353–15365. 30277767 10.1021/jacs.8b08933

[16] J. C. Theriot , C. H. Lim , H. Yang , M. D. Ryan , C. B. Musgrave , G. M. Miyake , Light. Science 2016, 352, 1082–1086. 27033549 10.1126/science.aaf3935

[17] A. M. Deetz , L. Troian-Gautier , S. A. M. Wehlin , E. J. Piechota , G. J. Meyer , J. Phys. Chem. A 2021, 125, 9355–9367. 34665634 10.1021/acs.jpca.1c06772

[18] L. Gao , Y. Zou , X. Liu , J. Yang , X. Du , J. Wang , X. Yu , J. Fan , M. Jiang , Y. Li , K. N. Houk , X. Lei , Nat. Catal. 2021, 4, 1059–1069.

[19] Y. Dong , T. Li , S. Zhang , J. Sanchis , H. Yin , J. Ren , X. Sheng , G. Li , M. T. Reetz , ACS Catal. 2022, 12, 3669–3680.

[20] S. Z. Sun , B. T. Nicholls , D. Bain , T. Qiao , C. G. Page , A. J. Musser , T. K. Hyster , Nat. Catal. 2023, 7, 35–42.

[21] Q. Zhou , M. Chin , Y. Fu , P. Liu , Y. Yang , Science 2021, 374, 1612–1616. 34941416 10.1126/science.abk1603 PMC9309897

[22] O. F. Brandenberg , R. Fasan , F. H. Arnold , Curr. Biotechnol. 2017, 47, 102–111. 10.1016/j.copbio.2017.06.005 PMC5617781 28711855

[23] O. F. Brandenberg , K. Chen , F. H. Arnold , J. Am. Chem. Soc. 2019, 141, 8989–8995. 31070908 10.1021/jacs.9b02931

[24] S. Dadashi-Silab , C. Preston-Herrera , E. E. Stache , J. Am. Chem. Soc. 2023, 16, 19387–19395. 10.1021/jacs.3c06783 37606469

[25] A. Simakova , M. Mackenzie , S. E. Averick , S. Park , K. Matyjaszewski , Angew. Chem. Int. Ed. 2013, 52, 12148–12151. 10.1002/anie.201306337 24115303

[26] T. Zhang , J. Yeow , C. Boyer , Polym. Chem. 2019, 10, 4643–4654.

[27] B. Zhang , X. Wang , A. Zhu , K. Ma , Y. Lv , X. Wang , Z. An , Macromolecules 2015, 48, 7792–7802.

[28] R. Li , Z. An , Angew. Chem. Int. Ed. 2020, 59, 22258–22264. 10.1002/anie.202010722 32844514

[29] S. Kumar , R. Gaddala , S. Thomas , J. Schumacher , H. Schönherr , Polym. Chem. 2024, 15, 2011–2027.

[30] R. Li , S. Zhang , Q. Li , G. G. Qiao , Z. An , Angew. Chem. Int. Ed. 2022, 61, e202213396. 10.1002/anie.202213396 36151058

[31] K. J. Rodriguez , B. Gajewska , J. Pollard , M. M. Pellizzoni , C. Fodor , N. Bruns , ACS Macro Lett. 2018, 7, 1111–1119. 35632946 10.1021/acsmacrolett.8b00561

[32] S. J. Sigg , F. Seidi , K. Renggli , T. B. Silva , G. Kali , N. Bruns , Macromol. Rapid Commun. 2011, 32, 1710–1715. 21842510 10.1002/marc.201100349

[33] A. Reyhani , M. D. Nothling , H. Ranji-Burachaloo , T. G. McKenzie , Q. Fu , S. Tan , G. Bryant , G. G. Qiao , Angew. Chem. Int. Ed. 2018, 57, 10288–10292. 10.1002/anie.201802544 29920886

[34] M. D. Nothling , H. Cao , T. G. Mckenzie , D. M. Hocking , R. A. Strugnell , G. G. Qiao , J. Am. Chem. Soc. 2021, 143, 293. 10.1021/jacs.0c10673 33373526

[35] M. R. Bennett , C. Moloney , F. Catrambone , F. Turco , B. Myers , K. Kovacs , P. J. Hill , C. Alexander , F. J. Rawson , P. Gurnani , ACS Macro Lett. 2022, 11, 954–960. 35819106 10.1021/acsmacrolett.2c00372 PMC9387098

[36] M. R. Bennett , P. Gurnani , P. J. Hill , C. Alexander , F. J. Rawson , Angew. Chem. Int. Ed. 2020, 59, 4750–4755. 10.1002/anie.201915084 31894618

[37] G. Fan , C. M. Dundas , A. J. Graham , N. A. Lynd , B. K. Keitz , Proc. Natl. Acad. Sci. USA 2018, 115, 4559–4564. 29666254 10.1073/pnas.1800869115 PMC5939106

[38] G. Fan , A. J. Graham , J. Kolli , N. A. Lynd , B. K. Keitz , Nat. Chem. 2020, 12, 638–646. 32424254 10.1038/s41557-020-0460-1 PMC7321916

[39] Y. H. Ng , F. Di Lena , C. L. L. Chai , Polym. Chem. 2011, 2, 589–594.

[40] K. Renggli , N. Sauter , M. Rother , M. G. Nussbaumer , R. Urbani , T. Pfohl , N. Bruns , Polym. Chem. 2017, 8, 2133–2136.

[41] A. P. Danielson , D. Bailey-Van Kuren , M. E. Lucius , K. Makaroff , C. Williams , R. C. Page , J. A. Berberich , D. Konkolewicz , Macromol. Rapid Commun. 2016, 37, 362–367. 26748786 10.1002/marc.201500633

[42] M. Daoud Attieh , Y. Zhao , A. Elkak , A. Falcimaigne-Cordin , K. Haupt , Angew. Chem. Int. Ed. 2017, 56, 3339–3343. 10.1002/anie.201612667 28194847

[43] A. P. Danielson , D. B. Van-Kuren , J. P. Bornstein , C. T. Kozuszek , J. A. Berberich , R. C. Page , D. Konkolewicz , Polymer 2018, 10, 741. 10.3390/polym10070741 PMC6403633 30960666

[44] A. Belluati , S. Jimaja , R. J. Chadwick , C. Glynn , M. Chami , D. Happel , C. Guo , H. Kolmar , N. Bruns , Nat. Chem. 2023 16, 564–574. 38049652 10.1038/s41557-023-01391-y PMC10997521

[45] T. B. Silva , M. Spulber , M. K. Kocik , F. Seidi , H. Charan , M. Rother , S. J. Sigg , K. Renggli , G. Kali , N. Bruns , Biomacromolecules 2013, 14, 2703–2712. 23739032 10.1021/bm400556x

[46] M. Zhu , S. Wang , Z. Li , J. Li , Z. Xu , X. Liu , X. Huang , Nat. Commun. 2023, 14, 1–10. 37328460 10.1038/s41467-023-39286-8 PMC10276049

[47] F. Zhou , R. Li , X. Wang , S. Du , Z. An , Angew. Chem. Int. Ed. 2019, 58, 9479–9484. 10.1002/anie.201904413 31067353

[48] J. A. Cowan , H. B. Gray , Inorg. Chem. 1989, 28, 2074–2078.

[49] A. W. Axup , M. Albin , S. L. Mayo , R. J. Crutchley , H. B. Gray , B. Science Washington , J. Am. Chem. Soc. 1988, 110, 435–439.

[50] S. Shanmugam , J. Xu , C. Boyer , J. Am. Chem. Soc. 2015, 137, 9174–9185. 26167724 10.1021/jacs.5b05274

[51] S. Shanmugam , J. Xu , C. Boyer , Macromolecules 2016, 49, 9345–9357.

[52] N. Sreerama , R. W. Woody , Anal. Biochem. 2000, 287, 252–260. 11112271 10.1006/abio.2000.4880

[53] A. C. Lehnen , J. A. M. Kurki , M. Hartlieb , Polym. Chem. 2022, 13, 1537–1546.

[54] C. A. Figg , J. D. Hickman , G. M. Scheutz , S. Shanmugam , R. N. Carmean , B. S. Tucker , C. Boyer , B. S. Sumerlin , Macromolecules 2018, 51, 1370–1376.

[55] L. Hub , J. Koll , M. Radjabian , V. Abetz , Polym. Chem. 2023, 14, 3063–3074.

[56] S. Shanmugam , C. Boyer , J. Am. Chem. Soc. 2015, 137, 9988–9999. 26171943 10.1021/jacs.5b05903

[57] M. Divandari , J. Pollard , E. Dehghani , N. Bruns , E. M. Benetti , Biomacromolecules 2017, 18, 4261–4270. 29086550 10.1021/acs.biomac.7b01313

[58] A. D. Adler , F. R. Longo , F. Kampas , J. Kim , Inorg. Chem. 1970, 32, 2443–2445.

[59] J. Kundu , U. Kar , S. Gautam , S. Karmakar , P. K. Chowdhury , FEBS Lett. 2015, 589, 3807–3815. 26606908 10.1016/j.febslet.2015.11.015

