# Supplementary material for: Catalyzing PET‐RAFT Polymerizations Using Inherently Photoactive Zinc Myoglobin
Source: Angew Chem Int Ed Engl. 2024 Oct 29;64(2):e202414431. doi: 10.1002/anie.202414431 (PMC11720391; doi:10.1002/anie.202414431)
Supplement: Supplementary file 1 — Supporting Information [file ANIE-64-e202414431-s001.pdf]

Angewandte Chemie
Eine Zeitschrift der Gesellschaft Deutscher Chemiker

Supporting Information

Catalyzing PET-RAFT Polymerizations Using Inherently Photoactive Zinc Myoglobin

*I. C. Anderson, D. C. Gomez, M. Zhang, S. J. Koehler, C. A. Figg**

Supporting Information for

Catalyzing PET-RAFT Polymerizations Using Inherently Photoactive Zinc Myoglobin

Ian C. Anderson, Darwin C. Gomez, Meijing Zhang, Stephen J. Koehler, and C. Adrian Figg*

Department of Chemistry and Macromolecules Innovation Institute, Virginia Tech, Blacksburg Virginia, 24061, United States of America

Experimental

Materials

Myoglobin (from equine skeletal muscle 95-100%, lyophilized powder), mesoporphyrin IX (95%), and 2-[[(2-Carboxyethyl)sulfanylthiocarbonyl]-sulfanyl]propanoic acid (99.0%) were purchased from Sigma Aldrich and used as received. *N*,*N*-Dimethylacrylamide (DMA, >99%) was filtered through basic alumina prior to use. All buffers and solvents were purchased from Thermo Fisher; buffer pH was monitored using a Thermo Fisher Orion Star A111 pH meter. ZnMIX was synthesized using the reported methods[1,2] and the purity was assessed by thin-layer chromatography and UV-Vis spectroscopy.

Analysis

^{1}H NMR spectroscopy was conducted on either an Agilent U4-DD2 400 MHz or a Jeol VH 400 MHz. Samples were prepared with D_2O (Cambridge Isotopes Laboratories, Inc., 99.9%) or $CDCl_3$ (Cambridge Isotopes Laboratories, Inc., 99.8%), which were used as received. ^{1}H NMR conversion was calculated using the equation $\%\,Conversion = \frac{[M_O]-[M_t]}{[M_O]} \times 100$ and the points on the pseudo first-order kinetics plot was calculated using $Ln\frac{[M_O]}{[M_t]}$.

Size Exclusion Chromatography (SEC) was performed in PBS with 200 mg/L NaN_3 at a flow rate of 0.5 mL min^{-1} (Agilent isocratic pump, degasser, and autosampler, columns: TOSOH TSKgel Guard PW_{XL} and TOSOH TSKgel G3000PW_{XL} molecular weight range 0-1 × 10^5 g mol^{-1}. Detection consisted of a Wyatt Optilab refractive index detector operating at 785 nm, a Wyatt DAWN multi-angle light scattering detector operating at 783 nm, and an Agilent MWD operating at 365 nm. Absolute molecular weights and dispersities were calculated with the Wyatt ASTRA software and off-line d*n*/d*c* analysis.

Visible light intensity was measured with an International Light Technologies ILT-350 illuminance spectrophotometer with a NIST traceable ISO17025 accredited calibration.

Circular dichroism (CD) spectroscopy was performed on a Jasco J-815 CD Spectrometer using a 1 mm pathlength Jasco quartz cuvette at room temperature. Samples were prepared at a concentration of 0.25 mg/mL in PBS.

UV-Vis spectroscopy was performed on a Cary 60 UV-Vis using 1 cm quartz cuvettes.

Procedures

Zinc myoglobin synthesis

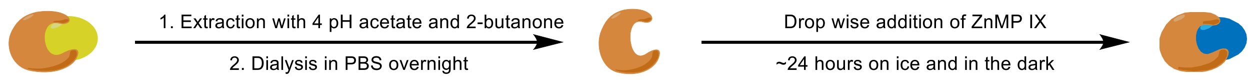

Apo myoglobin was prepared using a modified reported method.[1,3] Myoglobin (25 mg) was added to a 50 mL centrifuge tube and dissolved into 25 mL of acetate buffer chilled at 4 °C (pH 4) to yield a 1.0 mg/mL solution. An equal volume of butanone chilled at 4 °C (25 mL) was added to the tube and agitated to denature myoglobin and extract the heme group—the top organic layer was removed. This extraction process was repeated 3× until the organic layer was colorless. The solution was diluted 4× with PBS chilled at 4 °C, put into a 6-8 kDa dialysis bag (Spectra/Por 1 Dialysis Membrane Standard RC Tubing, SpectrumLabs Inc), and dialyzed against 5.0 L of PBS changing the dialysis buffer 3×. The solution was collected and centrifuged to remove any insoluble precipitates. The solution containing apo-myoglobin was concentrated using spin filtration with a 10 kDa MWCO spin filter (Pierce Protein Concentrator PES), until the volume was 100 mL. Separately, a solution of ZnMIX was prepared in PBS with 15 v/v% DMF using 6:1 molar equivalents of ZnMIX:apo myoglobin (determined using the 280 nm absorption peak, ε=13,980 L mol^{-1} cm^{-1}).[4] The ZnMIX solution was added dropwise to the apo myoglobin solution overnight in an ice bath. The ZnMb solution was purified using both CM 52 and G 25 size exclusion columns, concentrated using a 10 kDa MWCO spin filter (Pierce, Thermo Fisher) until the ZnMb concentration reached >3.0 mg/mL by UV-Vis spectroscopy (280 nm, ε= 13,980 ZnMb L mol^{-1} cm^{-1}). Solutions were used within 7 days of synthesis.

Procedure for DMA kinetics polymerizations

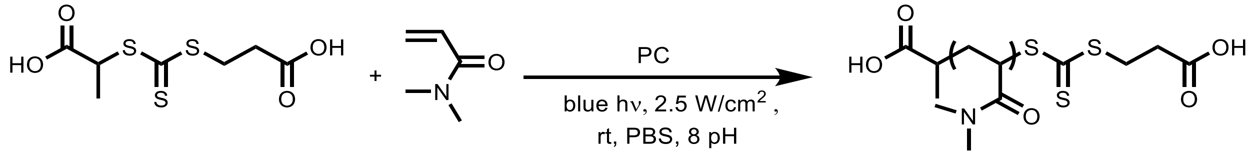

2-[[(2-Carboxyethyl)sulfanylthiocarbonyl]-sulfanyl]propanoic acid (6.4 mg, 0.025 mmol) was dissolved in a 2 dram vial in 2.0 mL of PBS using a heated sonicator bath. This solution was added to a Schlenk flask with DMA (0.48 mg, 5.0 mmol) and photocatalyst (2.5×10^{-4} mmol, solution concentration was 20 mg/mL for ZnMIX and variable for ZnMb). The reaction volume was brought to 5.0 mL using PBS to a [DMA] = 1.0 M. A final pH=8 was obtained using drops of 1.5 M NaOH and 1.0 M HCl solutions. The solution was bubbled with argon for 6 min, then irradiated with blue LED lights. Aliquots were periodically removed for ^{1}H NMR spectroscopy and SEC analysis.

Procedure for on/off studies

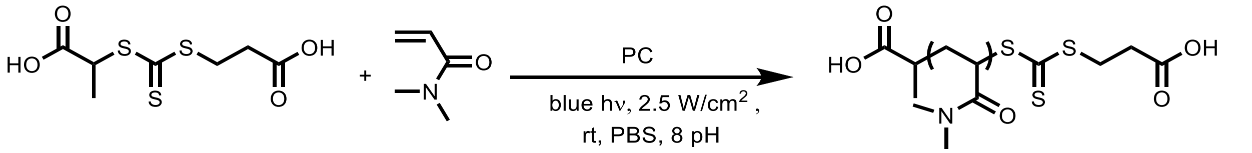

2-[[(2-Carboxyethyl)sulfanylthiocarbonyl]-sulfanyl]propanoic acid (6.4 mg, 0.025 mmol) was dissolved in a 2 dram vial in 2.0 mL of PBS using a heated sonicator bath. This CTA solution was added to a Schlenk flask with DMA (0.480 mg, 5.0 mmol) and photocatalyst (2.5×10^{-4} mmol, concentration varied between protein batches). The reaction volume was brought to 5.0 mL using PBS to a [DMA] = 1.0 M. A final pH=8 was obtained using drops of 1.5 M NaOH and 1.0 M HCl solutions. The solution was bubbled with argon for 6 min. The light was turned on or off for 1 h periods and a sample was taken at each time point for ^{1}H NMR spectroscopy.

Procedure for synthesis of poly(N,N-dimethylacrylamide) (PDMA macro-CTA)

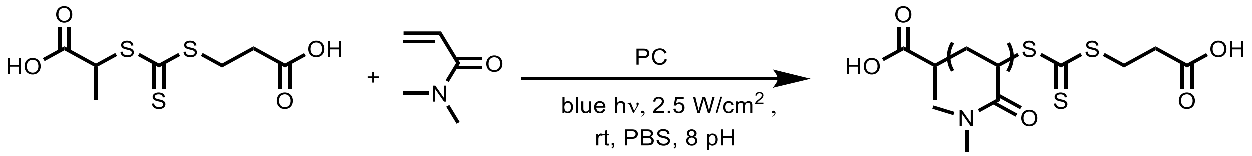

2-[[(2-Carboxyethyl)sulfanylthiocarbonyl]-sulfanyl]propanoic acid (0.10 mmol) was dissolved in a 2 dram vial in 7.0 mL of PBS using a heated sonicator bath. This solution was added to a 50 mL Schlenk flask with DMA (2.9 g, 30 mmol) and ZnMb (1.0×10^{-3} mmol, 3.8 mg/mL solution). The volume was brought to 30 mL using PBS with a minor amount of DMF as an internal standard. A final pH=8 was obtained using drops of 1.5 M NaOH and 1.0 M HCl solutions. The reaction was bubbled for 31 minutes using argon before being irradiated using blue LEDs. Aliquots of the reaction were taken to monitor DMA conversion by ^{1}H NMR spectroscopy until 37% monomer conversion was obtained. The reaction solution was quenched by opening the flask to air, diluted with ultra-pure water, put into a 6-8 kDa dialysis bag (Spectra/Por 1 Dialysis Membrane Standard RC Tubing, SpectrumLabs Inc), and dialyzed against water over 36 h, changing the water 2×. The dialyzed sample was centrifuged, decanted to remove any precipitants, and dried via lyophilization.

Procedure for PET-RAFT chain extensions of PDMA macro-CTA

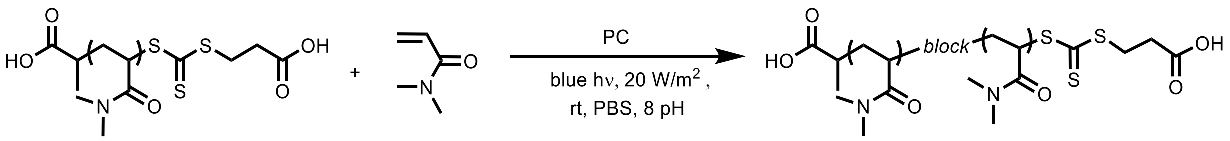

PDMA macro-CTA (140 mg, 0.10 mmol) was weighed into a 2 dram vial along with DMA (100 μL, 96 mg, 0.97 mmol), ZnMb (450 μL of 3.7 mg/mL solution, 1.0 × 10^{-4} mmol), and DMF as an internal standard. The final volume was adjusted to 3.0 mL. A final pH=8 was obtained using drops of 1.5 M NaOH and 1.0 M HCl solutions. The solution was bubbled with argon for 4 min and then irradiated with blue light. The ZnMb catalyzed chain extension ran for 16 h. The reaction was quenched by opening the vial to air and an aliquot was taken for ^{1}H NMR spectroscopy and SEC analysis.

Procedure for thermal chain extensions of PDMA macro-CTA

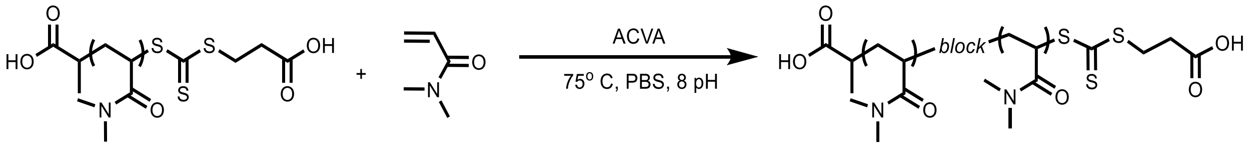

PDMA macro-CTA (140 mg, 0.10 mmol) was weighed into a 2 dram vial along with DMA (100 μL, 96 mg, 0.97 mmol), 10 μL of an ACVA solution (27.1 mg/mL), and DMF as an internal standard. The final volume was adjusted to 3.0 mL using PBS. A final pH=8 was obtained using drops of 1.5 M NaOH and 1.0 M HCl solutions. The solution was bubbled with argon for 4 min and then heated to 75 °C for 5 h. The reaction was quenched by opening the vial to air and an aliquot was taken for ^{1}H NMR spectroscopy and SEC analysis.

Additional Figures

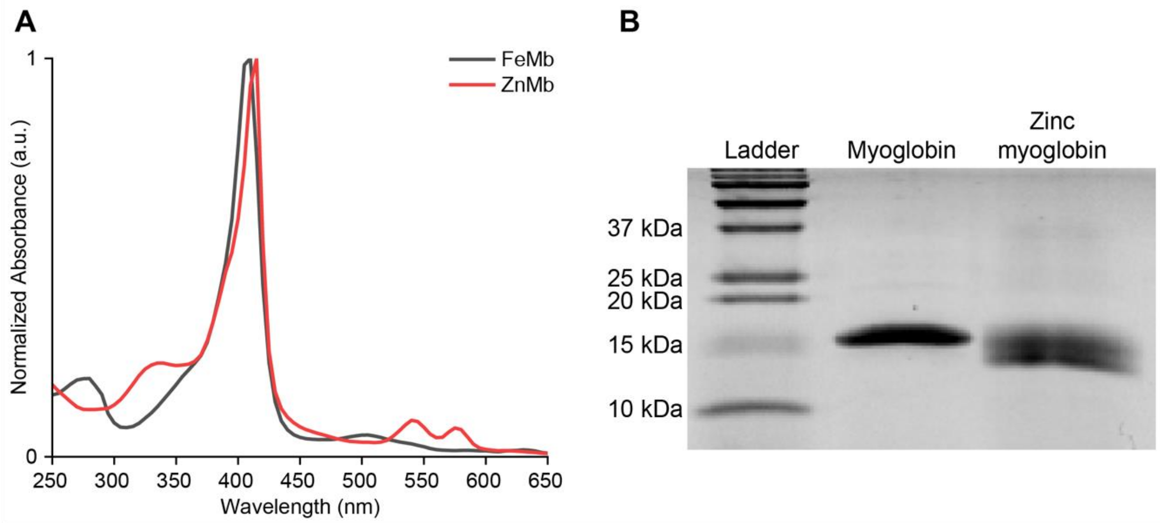

Figure S1. UV-Vis spectra (A) and SDS PAGE (B) of myoglobin (FeMb) and zinc myoglobin (ZnMb).

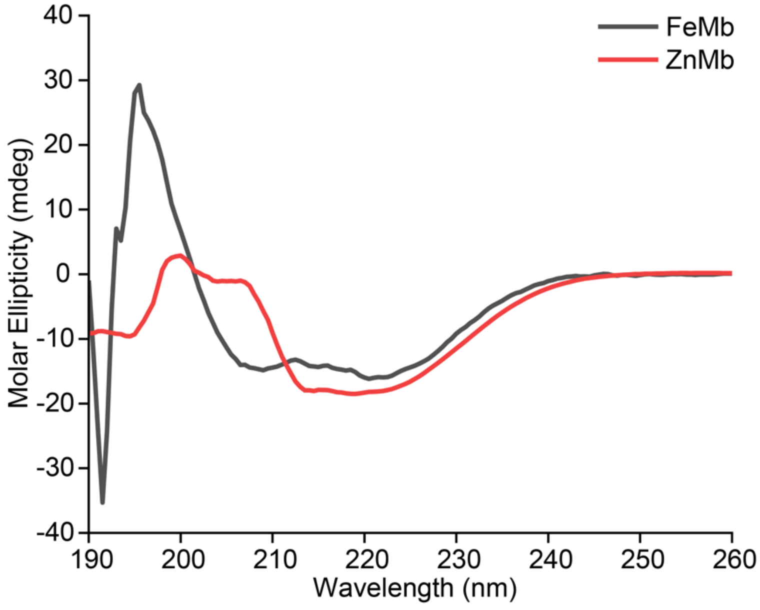

	**% relative change in secondary structure**
**regular α-helix**	+2.4%
**distorted α-helix**	-13%
**regular β-strand**	+10%
**distorted β-strand**	+8.1%
**turn**	+9.3%
**un-structured**	-9.4%

Figure S2. Circular dichroism spectra of myoglobin (FeMb) and zinc myoglobin (ZnMb) with CD Pro Analysis data detailing the changes to protein secondary structural features.

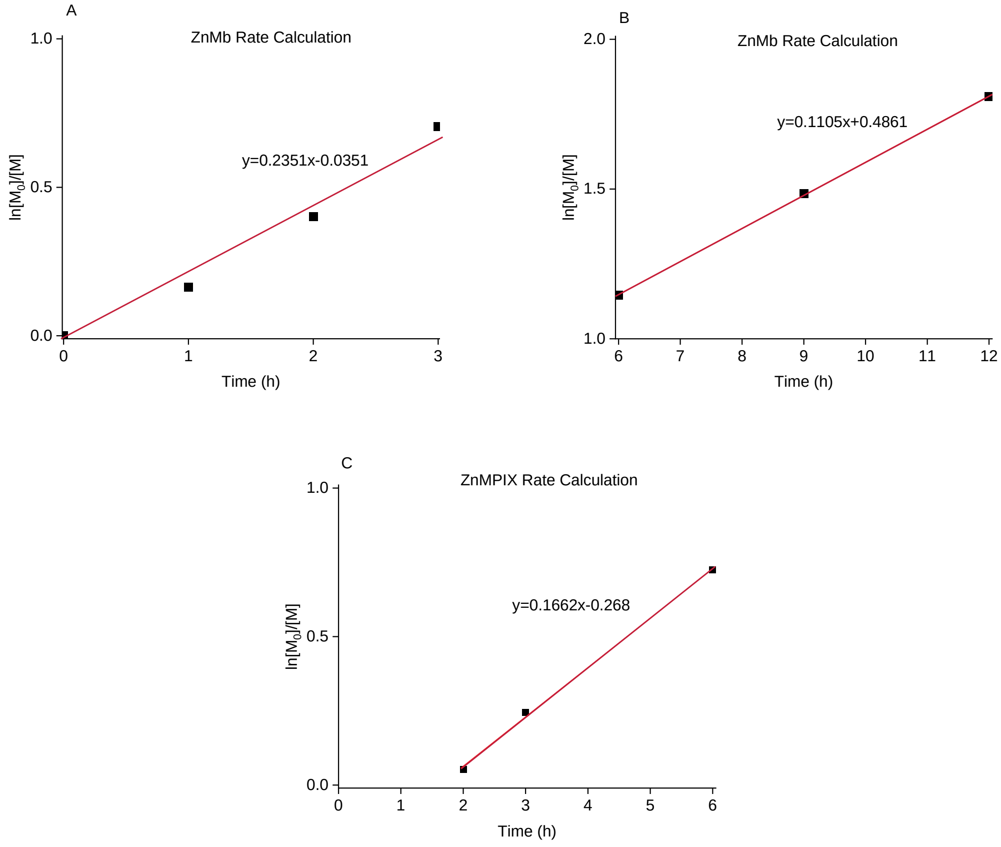

Figures S3. Apparent rate calculations from ZnMb and ZnMIX pseudo first-order kinetic plots. A. The first 4 time points (0-3 h) of ZnMb catalyzed polymerizations. B. The next 3 time points (6, 9, and 12 h) of of ZnMb catalyzed polymerizations. C. Rate calculation of 2, 3, and 6 h (chosen after the inhibition time) of ZnMIX catalyzed polymerizations.

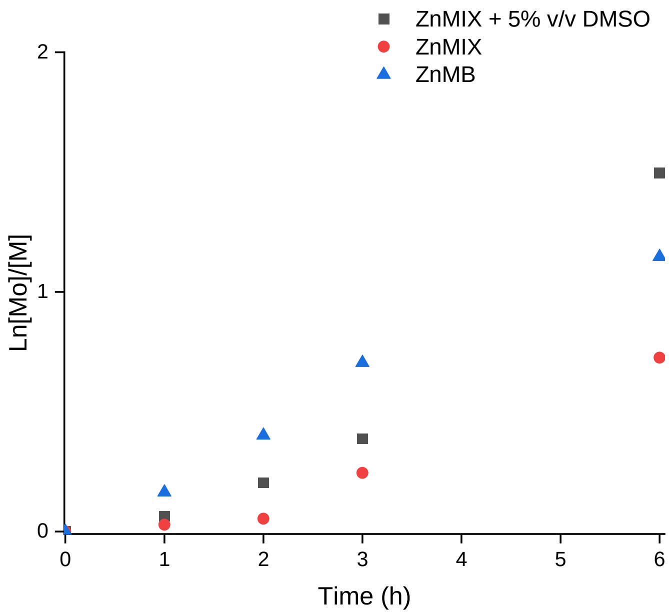

Figure S4. Comparison of DMA polymerizations catalyzed by ZnMb, ZnMIX, and ZnMIX with 5% v/v DMSO.

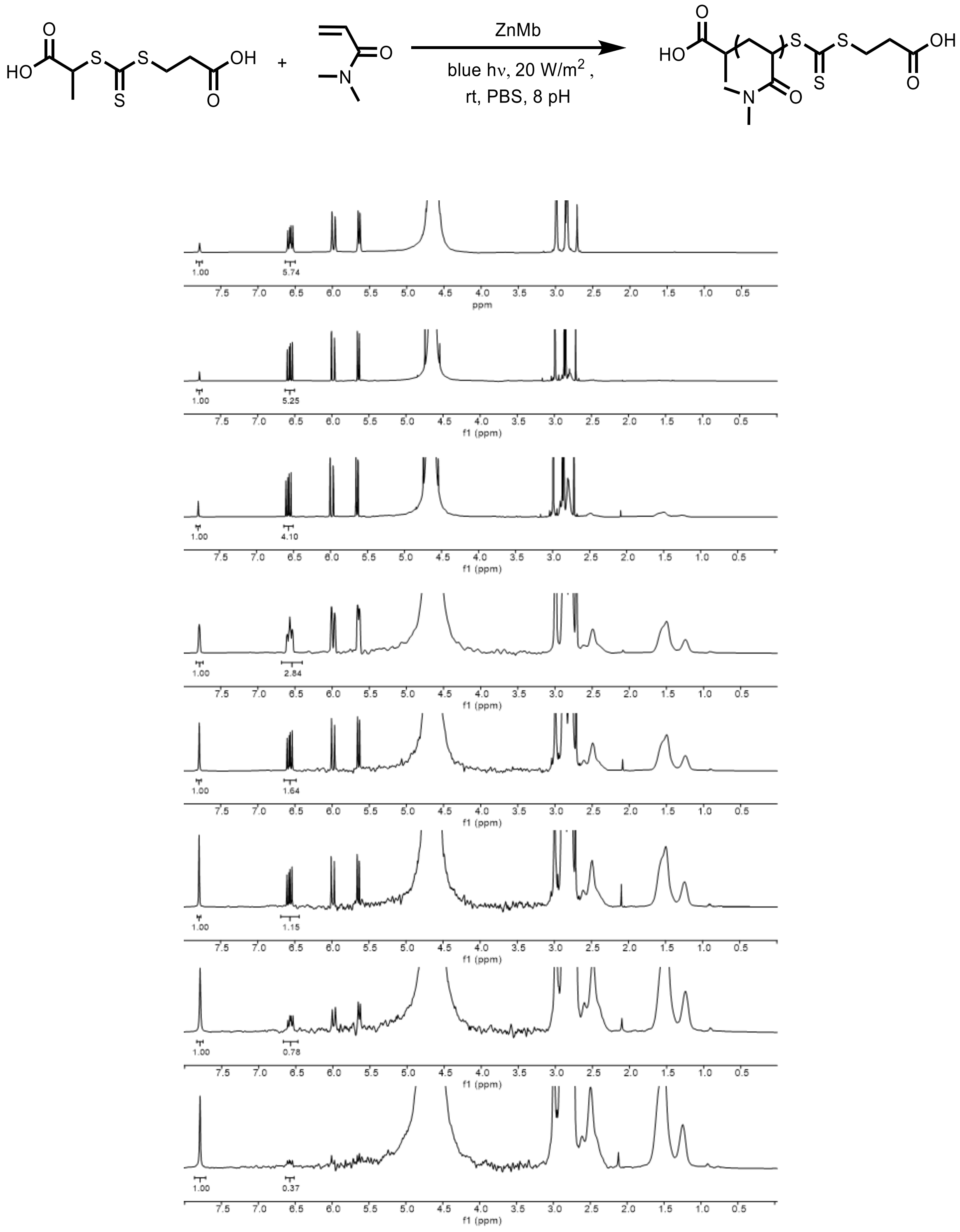

Figure S5. ^{1}H NMR spectra of example kinetics experiment of PET-RAFT polymerization catalyzed by zinc myoglobin monitoring the disappearance of a DMA vinyl protein (δ=6.5 ppm).

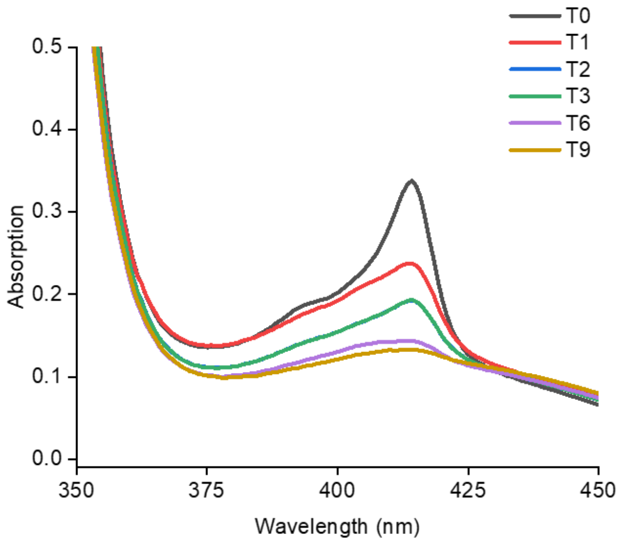

Figure S6. UV-Vis spectra measuring the absorbance of zinc myoglobin at time points 0-9 (T0-T9) during the polymerization.

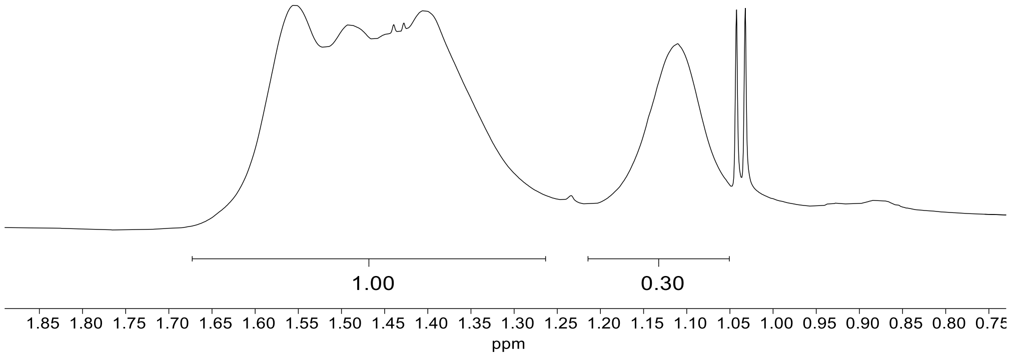

Figure S7. Tacticity analysis of PDMA sample using 600 MHz ^{1}H NMR spectrometer indicated that the synthesized polymers were atactic.[5]

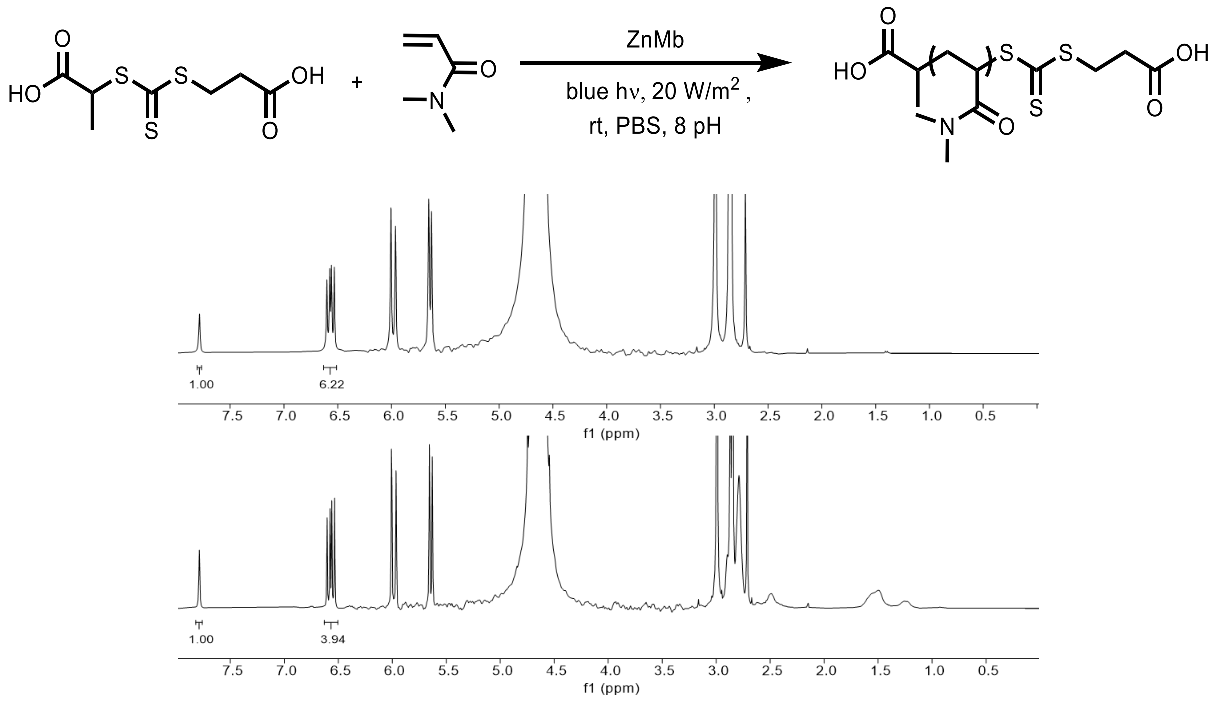

Figure S8. ^{1}H NMR spectra of the monomer conversion (37%) to synthesize poly(*N*,*N*-dimethylacrylamide) that was used for chain extensions.

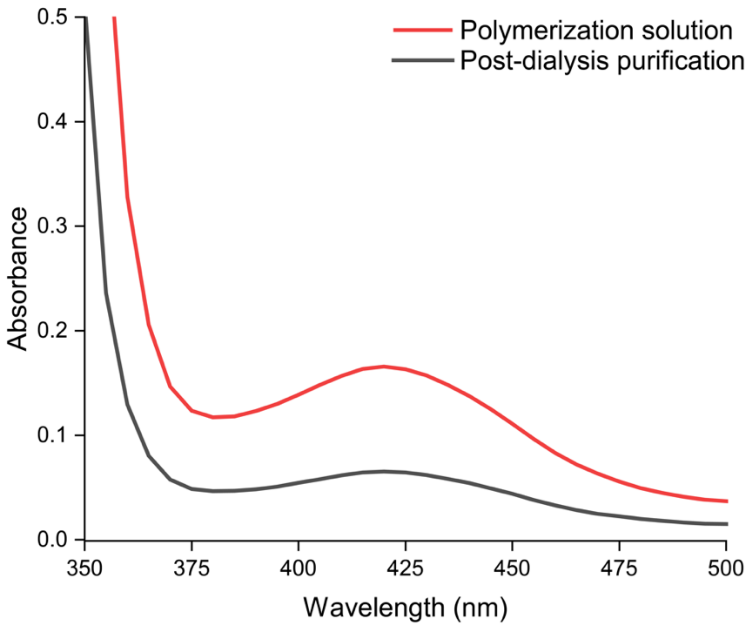

Figure S9. UV-Vis spectra measuring the removal of zinc myoglobin after dialysis purification of polymerizations.

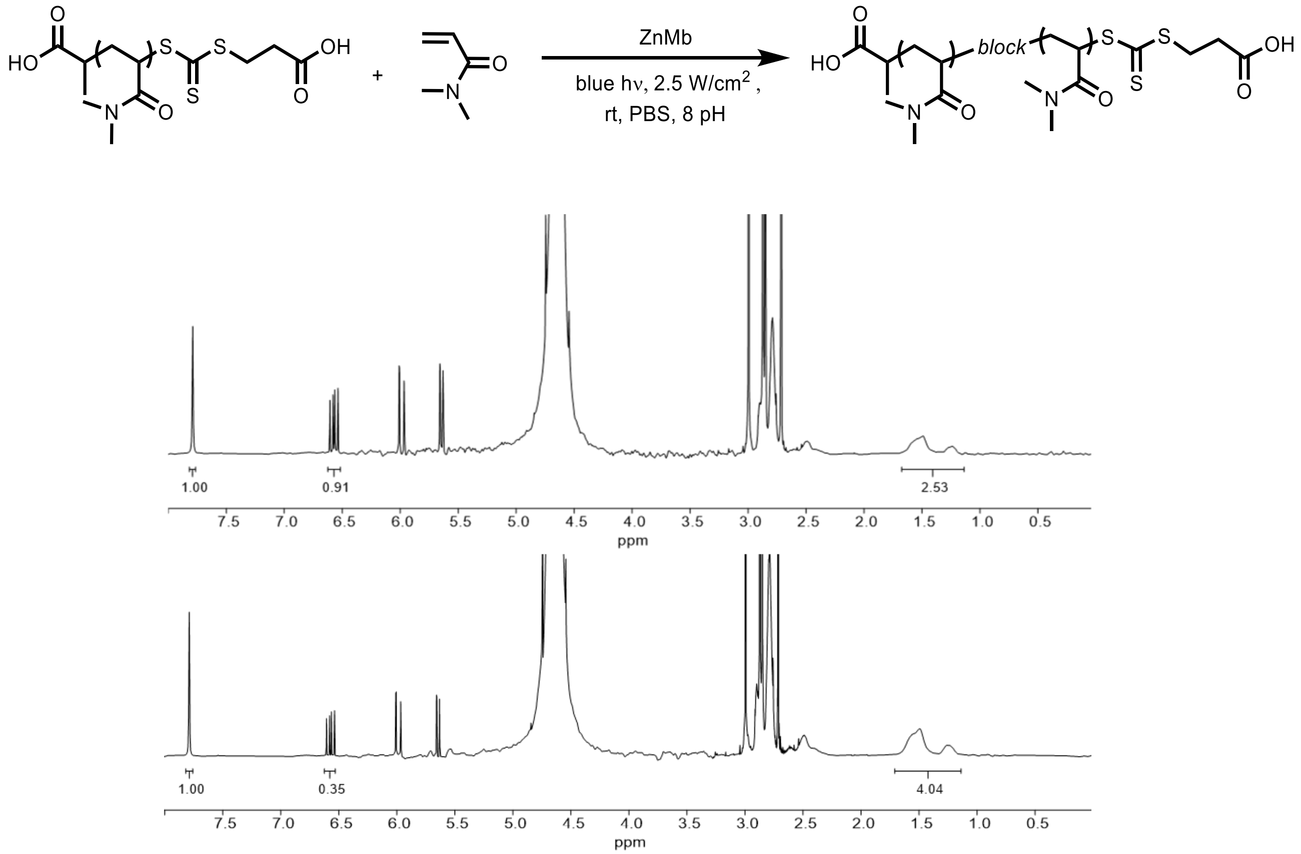

Figure S10. ^{1}H NMR spectra of the chain extension using ZnMb.

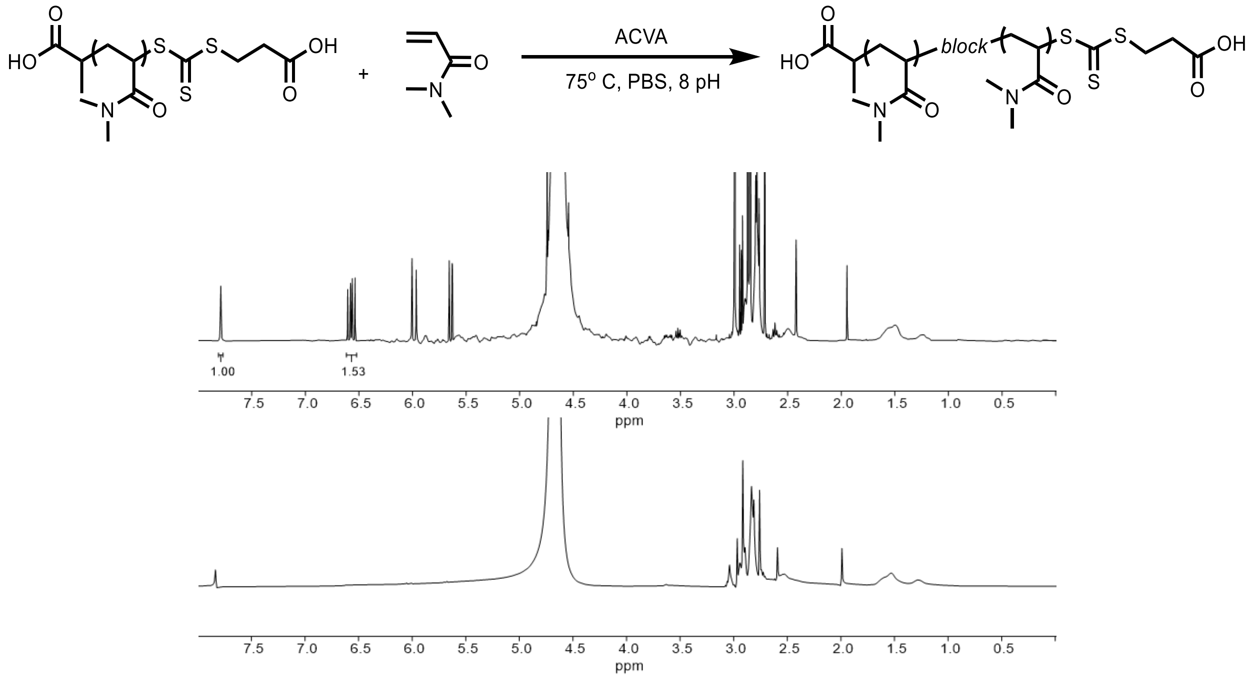

Figure S11. ^{1}H NMR spectra of the thermal chain extension using ACVA as an initiator

References

(1) Cowan, J. A.; Gray, H. B. Synthesis and Properties of Metal-Substituted Myoglobins. *Inorg. Chem.* **1989**, *28*, 2074–2078.

(2) Adler, A. D.; Longo, F. R.; Kampas, F.; Kim, J. On the Preparation of Metalloporphyrins. *J. Inorg. Nucl. Chem.* **1970**, *32*, 2443–2445.

(3) Axup, A. W.; Albin, M.; Mayo, S. L.; Crutchley, R. J.; Gray, H. B.; *Distance Dependence of Photoinduced Long-Range Electron in Zinc/Ruthenium-Modified Myoglobins*. *J. Am. Chem. Soc.* **1988**, *110*, 435-439.

(4) Kundu, J.; Kar, U.; Gautam, S.; Karmakar, S.; Chowdhury, P. K. Unusual Effects of Crowders on Heme Retention in Myoglobin. *FEBS Lett.* **2015**, *589*, 3807–3815.

(5) Shanmugam, S.; Boyer, C. Stereo-, Temporal and Chemical Control through Photoactivation of Living Radical Polymerization: Synthesis of Block and Gradient Copolymers. *J. Am. Chem. Soc.* **2015**, *137*, 9988–9999.
